# FiberGrowth Pipeline: A Framework Toward Predicting Fiber-Specific Growth From Human Gut Bacteroidetes Genomes

**DOI:** 10.3389/fmicb.2021.632567

**Published:** 2021-10-06

**Authors:** Bénédicte Colnet, Christian M. K. Sieber, Fanny Perraudeau, Marion Leclerc

**Affiliations:** ^1^Pendulum Therapeutics, San Francisco, CA, United States; ^2^Mines Paristech, Paris, France; ^3^Université Paris Saclay, INRAe, AgroParisTech, Micalis Institute, Jouy en Josas, France

**Keywords:** PUL, human gut bacteria, fiber, prebiotics, annotation, glycosyl hydrolase, growth prediction, Bacteroidetes

## Abstract

Dietary fibers impact gut colonic health, through the production of short-chain fatty acids. A low-fiber diet has been linked to lower bacterial diversity, obesity, type 2 diabetes, and promotion of mucosal pathogens. Glycoside hydrolases (GHs) are important enzymes involved in the bacterial catabolism of fiber into short-chain fatty acids. However, the GH involved in glycan breakdown (adhesion, hydrolysis, and fermentation) are organized in polysaccharide utilization loci (PUL) with complex modularity. Our goal was to explore how the capacity of strains, from the Bacteroidetes phylum, to grow on fiber could be predicted from their genome sequences. We designed an *in silico* pipeline called FiberGrowth and independently validated it for seven different fibers, on 28 genomes from Bacteroidetes-type strains. To do so, we compared the existing GH annotation tools and built PUL models by using published growth and gene expression data. FiberGrowth’s prediction performance in terms of true positive rate (TPR) and false positive rate (FPR) strongly depended on available data and fiber: arabinoxylan (TPR: 0.89 and FPR: 0), inulin (0.95 and 0.33), heparin (0.8 and 0.22) laminarin (0.38 and 0.17), levan (0.3 and 0.06), mucus (0.13 and 0.38), and starch (0.73 and 0.41). Being able to better predict fiber breakdown by bacterial strains would help to understand their impact on human nutrition and health. Assuming further gene expression experiment along with discoveries on structural analysis, we hope computational tools like FiberGrowth will help researchers prioritize and design *in vitro* experiments.

## Introduction

The human large intestine supports an extremely dense and diverse microbial community—up to 100 trillion individuals—known as the gut microbiota (Bäckhed et al., 2005). Over the last decade, the microbiome has been shown to play an important role in human health, and numerous studies have documented the link between microbiota composition and metabolic diseases, such as type 2 diabetes (T2D) (Qin et al., 2012), obesity, colorectal cancer (Thomas et al., 2019), immune response to treatment (Tanoue et al., 2019), and inflammatory bowel diseases such as Crohn’s disease (Henke et al., 2019), and also athletic performance (Scheiman et al., 2019). Microbial dysbiosis has been correlated to modern lifestyle, environmental parameters, medication, and western diet (Mosca et al., 2016). Indeed, one of the main drivers of microbiota composition has been shown to be diet, with long-term differences and fast responses to drastic diet changes, both in the metagenome and in the transcriptome (Filippo et al., 2010; David et al., 2013; Tap et al., 2015). One of the parameters playing a part in the booming number of individuals affected by metabolic disorders is the reduction of polysaccharide diversity in the day-to-day diet. As an example, migration from a non-Western country to the United States has been associated with immediate loss of gut microbiome diversity and function (Vangay et al., 2018). The mechanism of action being the depletion of dietary fibers—a nutrient category that includes a broad array of polysaccharides that are not digestible by human enzymes—in industrialized countries’ diet (Burkitt et al., 1972; Faith et al., 2011; Sonnenburg and Sonnenburg, 2014; Zmora et al., 2018).

However, the responses to a given diet are characterized by a large and not-yet-understood individual variability (Leshem et al., 2020) that complicates the design of specific diets or targeted foods and the understanding of glycan breakdown. The human microbiota produces complementary enzymes enabling the depolymerization and hydrolysis of dietary polysaccharides into sugars that can further be fermented into short-chain fatty acids (SCFAs). The enzymes completing this task, named carbohydrate-active enzymes (CAZymes), are involved in complex metabolic networks for the synthesis [glycosyltransferases (GTs)], degradation [glycoside hydrolases (GHs), polysaccharide lyases (PLs), carbohydrate esterases (CEs), and enzymes for the auxiliary activities (AAs)], and recognition [carbohydrate-binding module (CBM)] of all the carbohydrates on Earth. CAZymes are found in all living organisms (typically 1–3% of the gene content) and are particularly abundant (more than 3% of the gene content) in plants and microbes, e.g., *Bacteroides thetaiotaomicron* encodes for 391 CAZymes, which represent 8.2% of its genomic/gene content. In humans eating more fiber for 5 days, the expression of GH related to dietary fiber increased while mucus degrading GH were downregulated (Tap et al., 2015). Another argument supporting the link between CAZyme gene presence and strains’ metabolic capacities is the trend between CAZyme gene count and diversity, being almost a classifier of the bacterial genomes’ phylogeny (El Kaoutari et al., 2013). CAZyme gene expression was demonstrated *in vitro* using RNA-seq when strains were grown on specific polysaccharides (Rey et al., 2010; Martens et al., 2011; Scott et al., 2011). However, except for the Bacteroidetes phylum, there are still very few enzymatic systems being described and characterized. Annotating the glycosyl hydrolase genes and their loci organization/synteny is mandatory to characterize the human gut microbial capacity to breakdown glycans. Today, the CAZyme database of sequences and subfamilies is a reference for such genes. The database is built by an academic laboratory using manual expert editing of the annotation (Cantarel et al., 2008), while an open-source annotation tool called dbCAN is also available (Yin et al., 2012; Zhang et al., 2018).

Annotating CAZyme genes is not an easy task due to the modularity of the gene structure (Cantarel et al., 2008). Beyond difficulties to annotate CAZyme genes, the practical question to further investigate the link between the metabolic disorders (phenotype-level) and the genomic content of the microbiome is still an open question. Several publications reported that the CAZyme genes’ abundance in genomes could account for a richer metabolic network able to degrade different fibers or carbohydrates. Despite the link between specific GH and specific fibers documented by several authors (Zhao et al., 2018; Kovatcheva-Datchary et al., 2019), a consistent and complete list of CAZyme genes associated to a specific fiber is currently missing to any newcomer willing to understand the metabolic capacities of a strain from its genome sequence (as a starting point).

In addition to a complicated underlying link between carbohydrate breakdown and specific CAZy genes, taking into account the polysaccharide utilization loci (PUL) appears critical to understand the capacity of strains to hydrolase carbohydrates, in particular for the Bacteroidetes (Lapébie et al., 2019). Specifically, within the Bacteroidetes phylum, PULs are reported to be the genomic area that encodes the capacity to attach, degrade the fiber, and import oligomers. The term PUL was first coined by Bjursell et al. (2006) to describe clusters of colocalized, coregulated genes that contain functions such as detection, sequestration, enzymatic digestion, and transport of complex carbohydrates (Martens et al., 2009; Grondin et al., 2017). Indeed, the PULs encode a complement of cell surface glycan-binding proteins (SGBPs), TonB-dependent transporters (TBDTs), CAZymes (most frequently GHs and also PLs and CEs), and carbohydrate sensors/transcriptional regulators. Less reported in the literature, a similar structure has also been mentioned for Gram-positives (gpPULs) for butyrate-producing species belonging to the Firmicutes (Sheridan et al., 2015). Therefore, glycosyl hydrolases are encoded within operons that are not taken into account when simply annotating CAZymes. Currently, obtaining PUL annotation from a genome is not straightforward, and we found two available published resources. The PULDB database (Terrapon et al., 2017) of experimentally and non-experimentally proven PULs in Bacteroidetes is built as an extension to the CAZy database. The other one is a prediction tool called PULpy, identifying CAZymes that are co-localized with susCD gene pairs. The authors present their tool as a public version of the PULDB algorithm (Stewart et al., 2018). These current resources have drawbacks for non-experts: the first database has to be queried with either fibers or known species. Therefore, it is hardly usable for any new isolated strain. In addition, some interesting polysaccharides with prebiotic properties, such as inulin, are missing. The other tool could help since it provides an algorithm searching for hits similar to PULDB. However, the output is a prediction of a potential PUL with a number referring to the PULDB number and not specifically to a carbohydrate.

Taking this context into account, the goal of this manuscript is to attempt to bridge the gap between the microbiome metabolic capacities and strains’ genomes using a predictive model. Our approach is based on a simple microbiological standpoint where we consider the strains’ ability to grow on a specific carbohydrate as a measure of their metabolic capacities. This simple proxy allows us to have a simple experimental measure for which we suppose the specific genomic content could be predictive of such capacities. Hence, from a genome sequence, microbiologists could obtain a prediction of the metabolic capacities of a strain without having it grown on the fiber. The proof of principle is based on a benchmark data set that documented the growth differences of strains on different carbohydrates. We show that in taking PUL structures into account, our FiberGrowth tool improves growth prediction in comparison to only relying on CAZyme annotations of single genes.

## Materials and Methods

### Carbohydrate-Active Enzyme Annotation

Annotation of CAZymes was done using the open-source dbCAN2 pipeline (Yin et al., 2012; Zhang et al., 2018), which relies on three different algorithms: (1) hidden Markov models (HMMER) (Eddy, 2011), (2) alignment (DIAMOND) (Buchfink et al., 2015), and (3) peptide recognition (Hotpep) (Busk et al., 2017). The outputs are the genes associated with a CAZy gene based on each of the three algorithms. We chose to use the majority consensus rule: when a hit is found by two algorithms out of the three, then the gene is considered as a CAZy gene. As expected, the alignment algorithm (DIAMOND) provides more hits because of the modular structure of GH genes. In this manuscript, the pipeline dbCAN2 was used with the default settings.

In addition, we obtained CAZyme annotations from the CAZy database from B. Henrissat. The annotation was done in two steps: first, a BLASTP analysis of the predicted ORFs against the full-length sequences included in the CAZy database is performed (Cantarel et al., 2008). Second, the remaining sequences are manually analyzed by both (i) a BLAST search against individual GH, PL, CE, CBM, and GT modules and (ii) a HMMER3 search using hidden Markov models built for each CAZy module family. Raw CAZy annotations are presented in Supplementary Tables S1, S2. The strain selection was performed using the available growth dataset used in this manuscript.

### Glycoside Hydrolase Annotation Comparisons

The comparison between CAZy and dbCAN2 annotations was performed on 54 genomes on which 87 different GH families were screened (Supplementary Table S3) on the family level (e.g., the subfamily GH43_1 was considered as GH43 family).

### Growth Prediction Using Only Glycoside Hydrolase

To test how growth prediction performs with only one GH, we gathered such associations from four different publications (El Kaoutari et al., 2013; Park et al., 2018; Zhao et al., 2018; Kovatcheva-Datchary et al., 2019). The results are shown in Supplementary Figure S1.

### Building Fiber-Specific Polysaccharide Utilization Loci Models

**FIGURE 1:**
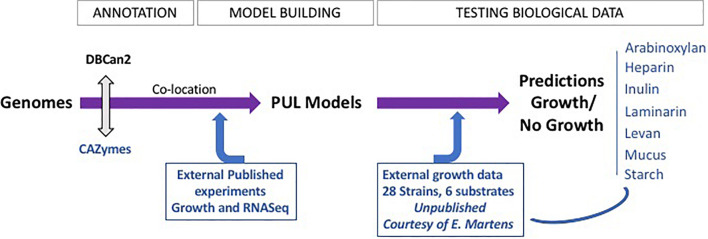
FiberGrowth pipeline strategy and proof of concept. The different steps of the pipeline are shown, highlighting the iterations between computing and integration of microbiology data. The PUL models, once designed, can then be used to process genomes within minutes. See Prediction of Polysaccharide Utilization Loci Using FiberGrowth in “Materials and Methods” section for additional pipeline specifications.

PUL models were constructed using CAZyme genes, publicly available gene expression data, and previously published data from growth experiments with RNA expression measurements (Figure 1). In the first step, we created candidate PULs based on previously published literature showing an association between CAZymes and fiber metabolism (Supplementary Figure S2; El Kaoutari et al., 2013; Park et al., 2018; Zhao et al., 2018; Kovatcheva-Datchary et al., 2019). We then refined the candidate PULs in analyzing available gene expression data from growth experiments with fiber-enriched media to refine the gene composition of PULs (Supplementary Table S4). Co-expressed neighboring genes of the candidate PULs were added, and genes without significant change in gene expression were removed. In addition, the genome sequence of 12 strains in combination with available growth data (Desai et al., 2016) was used to identify variations in gene composition and order for each PUL model. We then retrieved the gene family hidden Markov models (HMMs) for each gene of the PUL models from Pfam. If no Pfam annotation was available, we built a custom HMM by searching NCBI nr (Sayers et al., 2020) for orthologous genes. The retrieved amino acid sequences were aligned using ClustalW (default parameters) (Thompson et al., 1994) and HMMs built using HMMER3 (Eddy, 2011). The PUL model was tested against the genome from which it was inferred (majority of the PULs were inferred using the data on *B. thetaiotaomicron* DSM 2079), to obtain a positive control that the complete gene cluster was found. Because of the limited available RNA data, only seven PULs were built, for arabinoxylan, inulin, heparin, laminarin, levan, mucus, and starch (Table 1 and Supplementary Table S5 for genes included in each PUL).

**TABLE 1:** Performance of the FiberGrowth tool: the growth predictions for 28 genomes growing on seven fibers were compared to experimental data.

Fiber	True positive rate (TPR)	False positive rate (FPR)	Precision	Recall	Total_data
Arabinoxylan	0.89	0.00	1.00	0.89	28
Heparin	0.80	0.22	0.67	0.8	28
Inulin	0.95	0.33	0.91	0.95	28
Laminarin	0.38	0.17	0.75	0.38	28
Levan	0.30	0.06	0.75	0.3	28
Mucin	0.13	0.38	0.29	0.13	28
Starch	0.73	0.41	0.53	0.73	28

### Prediction of Polysaccharide Utilization Loci Using FiberGrowth

The FiberGrowth tool automatically predicts growth using fiber-specific PULs on a given bacterial genome. As input, it either takes a genome in fasta format or a gff file with gene locations together with their amino acid sequences in fasta format. If only a genome is provided, gene prediction will be performed using prodigal (Hyatt et al., 2010). In the next step, members of each fiber-specific reference PUL are identified using hmmscan of the HMMER package (Eddy, 2011). Based on the location and function, spatially clustered genes of carbohydrate active enzymes are determined by performing single linkage hierarchical clustering on the gene position using Euclidean distance. PUL candidates are retrieved by using a 5-kb threshold on the gene distance tree, allowing unannotated genes to be part of a candidate PUL. In the last step, candidates having all required core genes are reported (Supplementary Figure S0). Running the FiberGrowth tool on one bacterial genome takes about 1 min on a 2.3-GHz Intel Core i9 using one core. FiberGrowth is implemented in R (R Core Team, 2018) and makes use of the packages data.table (Dowle and Srinivasan, 2019), docopt (de Jonge, 2020), DT (Xie et al., 2020), gggenes (Wilkins, 2019), ggplot2 (Wickham, 2016, p. 2), knitr (Xie, 2015), magrittr (Bache and Wickham, 2014), rhmmer (Arendsee, 2017), rmarkdown (Allaire et al., 2020), and vroom (Hester and Wickham, 2020). The FiberGrowth code and PUL models are available on Github at https://github.com/wholebiome/FiberGrowth.

### Validation of FiberGrowth Pipeline With External Bacterial Growth Datasets

We compared the predictions of FiberGrowth to new external experimental measures of 28 bacterial strains’ abilities to degrade a wide variety of dietary and host-derived polysaccharides performed by Eric Martens (unpublished data, but kindly shared to benchmark FiberGrowth performance) and from a previous work (Desai et al., 2016; Supplementary Table S6). Briefly, these authors documented the growth of 534 strains including 28 types of strains, for which the genome sequence is available, on several polysaccharides and glycans as sole carbon sources (*n* = 2 replicate cultures per glycan substrate). Strains were grown on (i) a glucose-rich growth medium (PYG), (ii) a carbon-free minimum medium (PY), and (iii) a minimum medium with a polysaccharide as the only carbon source (PY + polysaccharide). The growth (OD_600 nm_) was recorded every 10 min when cultures were grown on PY + polysaccharide medium vs. PY medium only. The custom carbohydrate array was formulated according to Martens et al. (2011). We transformed the normalized growth results into a binary table filled with 0’s (no growth) and 1’s (growth) for each combination of strain and substrate (Supplementary Table S6). If the normalized growth was above 0.01, the strain was considered able to metabolize the substrate. Otherwise, the strain was considered not able to degrade the substrate. Then, the performance of the pipeline is used considering the predicted growth and the experimental ones using indicators such as the true positive rate (TPR—also called sensitivity) and the false positive rate (FPR), such as

$$\text{TPR}=\frac{N\,u\,m\,b\,e\,r\,o\,f\,t\,r\,u\,e\,p\,o\,s\,i\,t\,i\,v\,e\,p\,r\,e\,d\,i\,c\,t\,i\,o\,n\,s}{\begin{array}{c} N\,u\,m\,b\,e\,r\,o\,f\,t\,r\,u\,e\,p\,o\,s\,i\,t\,i\,v\,e\,p\,r\,e\,d\,i\,c\,t\,i\,o\,n\,s\;\\ +N\,u\,m\,b\,e\,r\,o\,f\,f\,a\,l\,s\,e\,n\,e\,g\,a\,t\,i\,v\,e\,p\,r\,e\,d\,i\,c\,t\,i\,o\,n\; \end{array}},$$

$$\text{FPR}=\frac{N\,u\,m\,b\,e\,r\,o\,f\,f\,a\,l\,s\,e\,p\,o\,s\,i\,t\,i\,v\,e\,p\,r\,e\,d\,i\,c\,t\,i\,o\,n\,s}{\begin{array}{c} N\,u\,m\,b\,e\,r\,o\,f\,f\,a\,l\,s\,e\,p\,o\,s\,i\,t\,i\,v\,e\,p\,r\,e\,d\,i\,c\,t\,i\,o\,n\,s\;\\ +N\,u\,m\,b\,e\,r\,o\,f\,t\,r\,u\,e\,n\,e\,g\,a\,t\,i\,v\,e\,p\,r\,e\,d\,i\,c\,t\,i\,o\,n\; \end{array}}.$$

The TPR and the FPR illustrate different properties of a predictor. For example if the goal is to find at least one strain growing on a substrate, a low TPR is a good characteristic. In other words, a predictor with a low TPR labeling a strain as growing ensures a good confidence on this prediction. But, if the goal is rather to not forget any strain that has a potential to grow on a strain, then a predictor with a high TPR is preferred.

## Results

### CAZy and dbCAN Annotation Comparison

**FIGURE 2:**
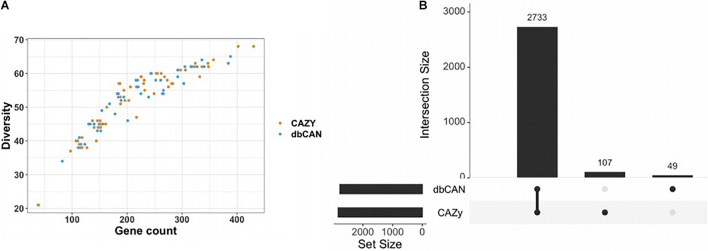
Number of common and different GH counts obtained by CAZy and dbCAN annotation methods. Annotations were performed on 54 different Bacteroidetes genomes on which the annotation was performed while screening for 87 different GH families. **(A)** A similar plot to El Kaoutari et al. (2013) highlighting a trend between the number of different GH family diversity and the GH total gene counts per genome. **(B)** A Venn diagram displaying, among all the GH predicted by dbCAN or CAZy, the number of common ones and different ones. Here, 2,733 genes were identically annotated by the two tools, while the manually curated CAZy brought 107 different annotations and dbCAN 49 others. Note that CAZy returned more GH genes than dbCAN.

Since two possible strategies exist to annotate glycosyl hydrolases, the automatic open-source pipeline dbCAN and a manually curated CAZyme database, we assessed the annotation differences between them. Fifty-four different Bacteroidetes genomes were analyzed, on which the annotations were performed while screening for 87 different GH families. The majority of the predictions regarding GH gene counts were identical and the diversity of the GH was evidenced by the large number of GH detected per genome, ranging from 100 to 400 (Figure 2A). However, the two annotation tools showed discrepancies. On a total of 2,889 genes detected on the 54 different genomes, 107 are only detected by CAZy and 49 only by dbCAN (Figure 2B). Beyond the gene count differences, it is noteworthy that, under our settings, some GH were not detected by one of the tools, and it depends on the GH family. For example, the CAZy detected GH24 and GH142 while dbCAN did not. Conversely, GH99 was annotated by dbCAN whereas CAZy was not, for this dataset. A detailed visualization is available on Supplementary Figure S3. The details of the GH families found by only one of the methods can be found in Supplementary Table S3. Using only the glycosyl hydrolase count to predict growth did not lead to meaningful results, with too many false positives (see Supplementary Figures S3, S4).

### FiberGrowth Prediction: Proof of Concept and Performance

**FIGURE 3:**
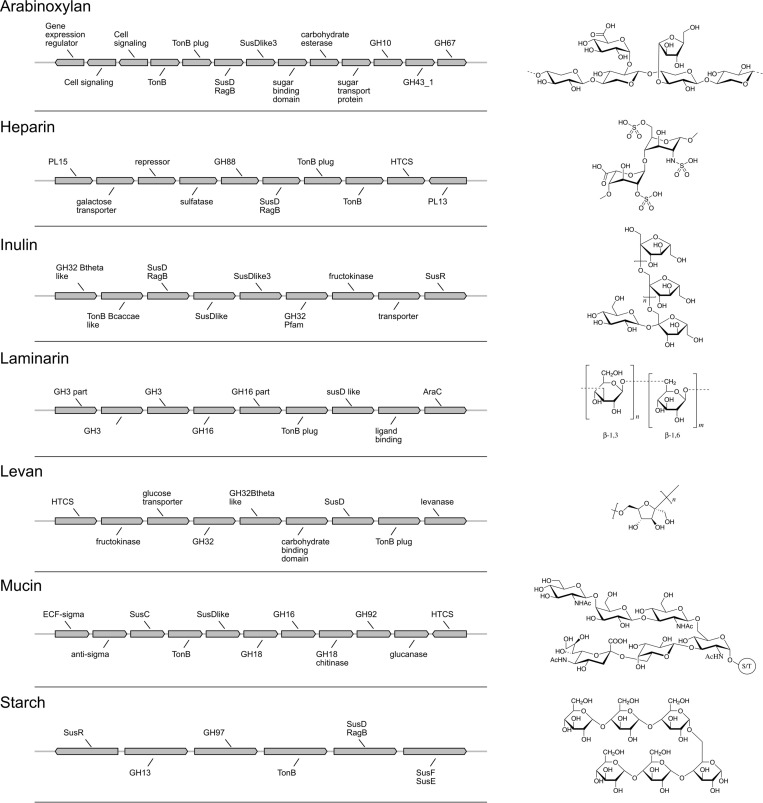
Polysaccharide utilization loci (PUL) gene models and fiber structures. Gene annotations and organization of each PUL model are shown on the left. Chemical structures of the associated fibers are drawn on the right.

We designed seven fiber-specific PUL models that are not specific to a certain genome and thus enable growth prediction on newly sequenced strains. The number of genes taken into account for a PUL model varies between 7 and 13, depending on the fiber (Figure 3 and Supplementary Table S5).

**FIGURE 4:**
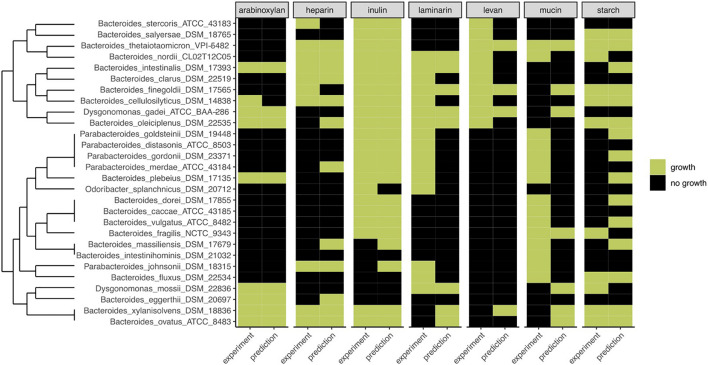
Comparison of FiberGrowth PUL-based predictions with experimental growth data for 28 strains from the *Bacteroidales* family. For each strain, the prediction and experimental result of growth (green) or absence of growth (black) on different growth media are shown. Dendrogram is calculated on experimental growth results.

To determine the predictive performance of our method, we compared the predictions with the experimental data for 28 strains, grown on PYG medium with or without fiber (Figure 4). One striking characteristic of the experimental growth data was that, besides inulin, very few strains grew on certain fibers, leading to table results with many 0’s. Furthermore, based on our preliminary 0.1 OD_600 nm_ threshold, for *Bacteroides intestinalis*, no growth at all was observed in the experimental data, and *Bacteroides vulgatus*, *Bacteroides caccae*, *Bacteroides massiliensis*, and *Bacteroides fragilis* only grew on one substrate. To better fit the experimental results, a 0.01 OD_600__–__*nm*_ threshold was used. Aware of these limits originating from the experimental data, we calculated FiberGrowth performance for each PUL model (Figure 4 and Table 1).

The best performance was measured for arabinoxylan with a TPR of 0.89 and FPR of 0. Only nine strains grew on arabinoxylan and only one false negative was detected (for *Bacteroides cellulosilyticus*). Our model, designed based on Martens et al. (2008), takes into account a total 10 genes from the operon, including the regulator of a two-component system (Figure 3). Here, the information is taken from Figure 2 (expression data for genes bacova_03417, bacova_03421-36, bacova_03437-40, and bacova_03448-50) and Supplementary Table S2 (gives more precisely the necessary three enzymes) with *Bacteroides ovatus* gene information. Note that in this reference, experimental data for *B. thetaiotaomicron* is reported with no growth and *B. ovatus* to grow due to the PUL expression, which is confirmed by the prediction (see Figure 4).

For heparin (TPR: 0.8; FPR: 0.22), the core genes GH88, PL15, and PL13 and a sulfatase are part of the model. Adjacent to it, the antisense PL13 is also part of the model and mostly adapted to high sulfate regions. Both PL13 and PL12, not included in the model, produce small oligosaccharides that only the exo-processive lyase PL15 is able to degrade. GH88 belongs to a family of enzymes that cleave the glycosidic linkage between the Δ4,5-unsaturated UA and GlcN/GlcNAc disaccharides.

The model for inulin showed the highest sensitivity (TPR: 0.95) with a FPR of 0.33. This was despite very opposite growth results compared to arabinoxylan, since 22/28 strains grew on inulin. The inulin PUL model (Figure 3) was built from Martens et al. (2011), using four protein-encoding genes: GH32, a fructokinase domain, a transporter, susC/susD homolog, and a susHT domain. The growth experiment RNA used was from *B. thetaiotaomicron*.

Levan, with a structure similar to inulin and a PUL composition that shared GH32 but included a specific levanase, led to not only a low sensitivity (TPR: 0.3) but also a low number of false positives (FPR: 0.06). The model accurately predicted growth for *Parabacteroides*, *Odoribacteriaceae*, and *Dysgonomonadaceae* but over-predicted growth for most *Bacteroides* genomes.

Performance of the mucin model was very low (TPR: 0.13; FPR: 0.38). The PUL model, inferred from gene expression data of strains growing on mucin, comprises 11 genes with GH18, GH16, and GH92. However, typical mucus-associated GH are missing (Figure 3). From a phylogenetic standpoint, it is worth noting that the model predictions were worse for the non-*Bacteroides* genomes, being wrong for all *Parabacteroides* genomes and *Dysgonomonas mossi*.

Laminarin (β1-3 and β1-6−glucan) found in brown algae is a glycan storage. The prediction performance was very low (TPR: 0.38; FPR: 0.17) based on the genes selected for the PUL. Similarly to mucin, it is noteworthy that the predictions were all wrong for the eight non-*Bacteroides* genomes, which all grew on this substrate.

**FIGURE 5:**
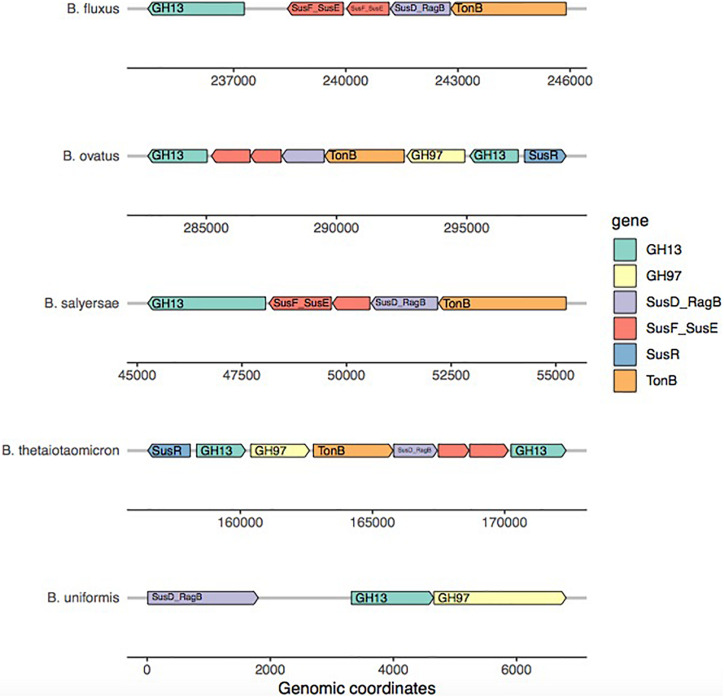
Amylase PUL visualization across five *Bacteroides* genomes. For each strain, only the most complete PUL is represented. The reference to build the starch model came from *Bacteroides thetaiotaomicron*. This representation highlights the lack of synteny of the starch PUL within the genomes of strains belonging to the same genus.

Surprisingly, the starch model led to a high sensitivity (TPR: 0.75) with a FPR of 0.41 as trade-off. This PUL model was designed using a widely recognized set of genes, consistent among the literature: the amylase GH13 and GH17 and the susD-RagB, susF-SusE, susR, and TonB. The model was trained using Bt transcriptomics data. However, the visualization of the new PUL shown in Figure 5 highlighted the lack of synteny of the starch PUL between closely related genomes. The model wrongly predicted growth of *Bacteroides dorei*, *B. intestinalis*, *B. massiliensis*, *Bacteroides plebeius*, *B. vulgatus*, *Parabacteroides goldsteinii*, and *Parabacteroides gordonii*. On the opposite, it did not predict growth of five strains *B. fragilis*, *Bacteroides fluxus*, *Bacteroides nordii*, *Bacteroides salyersae*, and *Dysgomonas mosslii.*

Some insights can be obtained by taking into consideration the taxonomic differences of the genomes analyzed. From the 28 genomes belonging to the *Bacteroidales* order, eight were from different families than the *Bacteroidaceae*. For the five *Parabacteroides* genomes analyzed, the model accurately predicted growth on inulin for all of them and also on levan, heparin, and starch for each of the two strains growing on it. The five strains grew on laminarin and mucin, but the model missed those five positive growth results. *Parabacteroides johnsonii* appeared separated on the clustering dendrogram because of its results on starch and growth on heparin but no growth on inulin. Interestingly, for the less studied *Dysgonomonas* genus (*Dysgonomonadaceae*), the strains *Dysgonomonas gadei* and *Dysgonomonas mossii* grew on three and four fibers and were accurately predicted, except again for mucin for *G. gadei* and for starch for *D. mossii*. Finally, *Odoribacter splanchnicus* (*Odoribacteraceae*) did not grow on any substrates except inulin and laminarin, and both were wrongly predicted by the model.

## Discussion

In this manuscript, we designed a pipeline to predict the growth of Bacteroidetes species from the human gut on seven different polysaccharides using a combination of *in silico* modeling and validation with microbiology data. To our knowledge, our work provides the first integrated pipeline to use PUL to investigate the growth of human gut strains on specific polysaccharides.

PUL models have already been described by Terrapon et al. (2015), but the approach is different, centered on specific genomes to provide a unique model based on previous biochemical characterizations of the enzymes and proteins involved. For taxonomic assignment and phylogenetic placement of existing GH or new GH, the SACCHARIS pipeline automatically annotates GH and provides accurate phylogenetic functional trees (Jones et al., 2018).

Our hypothesis was that, by using growth and transcriptomics data from the literature, new fiber-specific PUL models could be built and assessed on a distinct growth data set. Hence, the integrated pipeline, including a new automated annotation of PULs, could provide microbiologists, in a minute, with growth predictions from genome sequences. The pipeline showed very different accuracies, depending on the fiber, from excellent, up to 96%, to 38% for mucin, close to a random association.

Despite building a PUL model that followed a good agreement within the scientific community for the GH and associated genes involved in starch hydrolysis, and using available transcriptomics data to build the model, it performed poorly compared to the other models. Anderson and Salyers (1989) first reported in the late 80s that the breakdown of starch by *B. thetaiotaomicron* involved outer membrane bound-attached starch-binding sites and periplasmic starch-degrading enzymes, rather than only extracellular enzymes. Since then, starch catabolism has been largely characterized, and Foley et al. (2016) described the Sus operon as the model system for starch uptake in Bacteroidetes. The complexity and substrates’ diversity of starch-related polysaccharides hardly fit the CAZyme database. GH13 displays a wide phylogenetic diversity (as described by Stam et al., 2007) that is now classified under GH13 subfamilies in CAZyme. The visualization of the genes’ organization of our PUL models for few *Bacteroides* genomes is consistent with the large diversity and lack of synteny between close genomes. In the *in vitro* experiments we considered, starch was from potato starch (Eric Martens’ data), and the results would be different from a different origin or amylose/amylopectin ratio. Furthermore, starch can be found under distinct biochemical structures, in *in vitro* experiments (RS2 or RS3) and in food: not only starch as found in fruits but also the different resistant starch structures, formed after cooking and cooling down starchy food, or chemically processed resistant starch (RS4). In humans and in pigs, the microbial community composition was found to be linked to the starch structure, emphasizing that variability can be explored and understood only through the use of starches with highly characterized structures (Warren et al., 2018). It is then possible to speculate that other uncharacterized operons for starch breakdown exist in Bacteroidetes genomes, and, as a matter of fact, we detected incomplete PUL, where annotations are missing. Furthermore, starch is a carbohydrate storage in plants, hence, relative to the global evolution, has not been consumed as cooked by humans until recently. Whether or not this large diversity of amylase systems reflects recent evolution remains to be determined. It is worth noting that in other bacterial phyla, genomic systems described for the breakdown of starch differ from the common set of genes typically reported and used in our model. It has been recently demonstrated that in *Ruminococcus bromii* L2-63 a cell surface amylosome and sporulation capacity exist for starch breakdown in strains from the human and rumen microbiota (Mukhopadhya et al., 2017).

Laminarin is also a storage glucan and the growth prediction was low. The performance was similar to the one for starch, despite a very different situation: our PUL model was inadequate since, based on the methodology and annotation, using GH3 and GH16, we might not have found an appropriate marker GH. However, a seven-gene PUL has been described for *Bacteroides uniformis*, which includes CBM6/GH3, GH158, and GH16.

Devillé et al. (2007) reported that laminarin modulated the microbiome and increased propionate and butyrate in fermenters, pointing toward an effect on not only Bacteroidetes but also Firmicutes. Furthermore, it modulated mucus composition in rats’ gastrointestinal tract (Devillé et al., 2007). It was recently shown by another research group (Strain et al., 2019) that an addition of laminarin into digesters increased *Lachnospiraceae*, compared to fructooligosaccharide (FOS) and cellulose. The mucin model led to the lowest prediction performance. Interestingly seven out of the eight non-*Bacteroides* genomes led to a false prediction. It was built using PULDB and included 11 genes, but several key GH involved in mucin breakdown were not accounted for. The PUL model was based on transcriptomics data, and because of the complexity of mucin, the GH genes over-expressed at this time point were limited. Another issue is cDNA annotation. For instance, one of the main causes of misannotation of the *fuc* genes is their similarity to the genes for rhamnose utilization. Both the FucK and RhaB proteins belong to the FGGY family of carbohydrate kinases (Pfam: PF02782).

Mucin used in experiments is very frequently pig gastric mucin type III. However, mucin composition depends upon the individual and the GI tract segment, and its complexity has only recently been acknowledged. Mucin is at a crossroad between dietary substrates and human secretion. Indeed, using droplet microfluidics, we recently demonstrated and characterized a new GH enzyme, active on human gangliosides, with similar structure to human mucin and milk oligosides and overrepresented in IBD patients’ metagenomes (Tauzin et al., 2020). The mucin composition and degradation pathways still remain to be elucidated.

Interestingly, the link between mucin and heparin has been shown *in vivo*: the expression of heparin PUL in mice colonized with *B. thetaiotaomicron* (*B. theta*) could only be observed in bacteria occupying the mucosal layer of the gastrointestinal tract, suggesting that *in vivo* mucus could be a source of heparin *in vivo* (Li et al., 2015). Furthermore, GH88 included in our heparin PUL model plays a key role in bacteria–mucus interaction: when mice were co-colonized with six other Bacteroidetes strains in addition to the *B. theta* mutant, the *B. theta* GH88–mutant was much lower in abundance than in mono-colonized mice, indicating that the ability to degrade heparin is under increased selection pressure for *B. theta* in the presence of other Bacteroidetes. Heparin is an interesting glycan: it is an anticoagulant drug, with a structure of the glycosaminoglycan family of carbohydrates, bearing similarities with mucin, since the most common disaccharide unit is composed of a 2-O-sulfated iduronic acid and 6-O-sulfated, N-sulfated glucosamine. As a matter of fact, heparins from most commercial preparations originate from beef lung or porcine intestinal mucosa. Our model led to a good growth prediction, and interestingly, *a posteriori*, we had obtained a PUL structure very similar to the one built by Joglekar et al. (2018). The authors documented the complex synteny for heparin PULs within the *Bacteroides* genus: for example, in *Bacteroides eggerthii* and *Bacteroides galinarium* genomes, another heparin PUL exists, with PL15 but not PL13. The prediction from our model could be expected to be low for strains that do not encode for a PL13 or PL15, such as *B. eggerthii* DSM20697, *B. galinarium* DSM 18171, *B. stercoris* ATCC 43183, and B. YIT 12058. Because the human epithelium contains high amounts of heparin sulfate, the biologically relevant glycan PUL is heparin sulfate, hence with PL13 as a priority gene to target. We did not include GH95, involved in mucin degradation, because its α-L-fucosidase activity is only found in very few heparin PULs, in some *B. thetaiotaomicron*, *B. ovatus*, and *B. finegoldii* genomes.

Growth prediction for inulin shows good performance. FOS are short-chain oligosaccharides that are generated by hydrolysis of the polysaccharide inulin, which is composed of 2–60 fructose monomers. We presented the results of inulin and levan next to each other because they represent two distinct glycosidic linkages (2–6 in levan and 2–1 in inulin) that are present in the fructan homopolymers and that are available to the gut microbiota. Inulin is found in different nutrients such as wheat, onion, garlic, and banana and is the most common used fiber in prebiotics that, when used in combination with other probiotics, is able to promote the growth of specific beneficial gut bacteria such as bifidobacteria (Gibson et al., 1995). GH91, an inulin lyase, has been described as involved in the hydrolysis of inulin. Interestingly, while GH32 appears to be always necessary, our results showed that GH91 is not. A close examination of GH91 indicates that the enzyme activity releases difructofuranosyl 1,2 23 diamyhide that seems kept within the cytoplasmic compartment, consistent with the absence of a signal peptide on the gene sequence (Henrissat, personal communication). The absence of release of fructose and the location of the enzymatic activity seem to indicate that GH91 is not mandatory and that its role might not be on catabolism but potentially for intra-cytoplasm metabolism or storage.

Recent work by the group of Joglekar et al. (2018) on Bt strains VPI-5482 (same strain as used in our study) and Bt-8736 contrasted levan and inulin or fructan operon with GH phylogenetic trees. They demonstrated that related genetic loci can encode diversified biochemical pathways in strains from the same *B. thetaiotaomicron* species. The presence of GH32, SGBP, and SusD and SusC-like domain, corresponding to outer membrane binding proteins, explained the capacity to grow. Accordingly, in our growth prediction model, *B. finegoldii*, in the same phylogenetic cluster as Bt VPI-5482 for the SusC and SusD, does not grow on inulin. Furthermore, it has been demonstrated that the presence of the divergent susC/susD gene alone enabled the hybrid Bt(8736-2) strain to outcompete the wild-type strain *in vivo* in mice fed an inulin diet. This pathway does match our model, which includes enzymes and carbohydrate-binding and import proteins with distinct substrate specificities, which could not have been predicted previously based on sequence data alone.

The discrepancy between databases regarding GH annotation can be highlighted in the case of levan PUL, looking at the domain level. Our results showed that taking into account three GH32 distinct gene copies and a levanase within a PUL model was sufficient to predict growth on levan, but the model over-predicted growth in *Bacteroides* genomes. However, the PUL was built using transcriptomics data, which raises questions on cross-annotation of cDNA and genomes. Indeed, Pfam annotation from the transcriptome provided an N-terminal domain for the GH32 used in this model, instead of a catalytic domain. One can predict that the catalytic domain from Pfam would provide a better performance. Recent work brought some insight onto the PUL related to inulin or levan metabolism. A closer look at the inulin/levan- or fructan-associated operon recently described by Joglekar et al. (2018) confirmed that the specificity for the 2-6 linkage found in levan is from a GH32 cell surface endo-levanase and an ortholog of BT1761, a surface glycan-binding protein. The presence of the cell surface levanase of *B. thetaiotaomicron* VPI-5482 was critical for the ability of this strain to use the levan. The authors very elegantly demonstrated how structural differences present in dietary polysaccharides such as fructans can result in distinct molecular mechanisms for utilization of these polymers.

Despite obtaining low performance for some fibers, the overall goal of this analysis is a metabolic functional assessment of the different strains. We also tested whether an approach involving the use of GH genes only would be sufficient to obtain a growth prediction. Several teams applied the following method: gathering enzymes into functional groups, for example GH23, GH25, and GH73 being dedicated to peptidoglycan breakdown and GH13 is dedicated for starch breakdown. A first drawback in implementing this method into a pipeline is that the attribution of a function or the link between GH and a substrate or fiber varies substantially across the literature. For instance, we gathered such associations from four different publications (El Kaoutari et al., 2013; Park et al., 2018; Zhao et al., 2018; Kovatcheva-Datchary et al., 2019) and found that discrepancies can be observed. For example, GH95 being either associated with mucin degradation or with cellulose degradation depending on the publication. Supporting our PUL approach, using GH only led to growth predictions that were not meaningful.

Several parameters play a part in the model prediction performance. Both annotation quality and discrepancy between datasets and gene and enzyme terminology differences have been a hurdle in designing new PUL models. As shown by the comparison of GH annotation tools, an accurate annotation of CAZymes is key to improve prediction performance. Most automated annotation pipelines for transcriptomic data do not accurately annotate for GH. Then, the Pfam domain used for functional domain characterization may easily provide the right annotation of only one domain of the GH, as we observed for several PUL where we did not capture the GH catalytic domain. Another aspect is that our PUL models do not have a size limit, as long as the distance between neighboring genes annotated as the same PUL family is less than 5 kb. This is consistent with large syntenies observed in PULDB. For example, for our PUL amylase model, *B. uniformis* PUL is slightly over 6 kb while *B. salyersae* PUL reached 10 kb.

False prediction may originate from several reasons including *in vitro* conditions or how the model processes growth data. The PUL models were obviously sensitive to the growth/OD threshold. For arabinoxylan, the three strains predicted “no growth” by the model and grew were *B. massiliensis*, *B. oleiciplinus*, and *B. merdae*. Interestingly, in the four cases where the model predicted “no growth” and growth was counted positive based on the model threshold, the OD_600__–__*nm*_ recorded were below 0.1 (*B. intestinalis*, 0.05; *B. johnsonii* and *B. clarius*, 0.06; and *B. finegoldii*, 0.14). A similar phenomenon happened for heparin, and a reset growth threshold improved performance. The growth threshold could be adjusted accordingly for the different fibers, when more experimental data are available. However, the experimental growth medium itself influences growth yield in a strain-to-strain manner that is difficult to predict. The results would need to be reassessed or taken with caution if strains were grown in a culture medium that is different from the reference. The experimental data we used to measure the pipeline performance were obtained in PY medium. However, the transcriptomics data used to build the PULs models were obtained on CM medium, which could account for differential genes being over-expressed, at the time of sampling, compared to growth validation data on PY medium. Indeed, the growth medium may change which genes are prioritized in the model. It remains to be determined whether this bias would be more impactful on complex PUL models with large numbers of genes or complex operon structure where genes, not co-transcribed, might not be captured in a single transcriptome time point. The extreme modularity of some PULs indicates that the conclusions about growth results from one species to another have to be taken with caution since, like others, we detected strain-to-strain variations within the same species for inulin and levan.

The complexity of fiber structures makes the links between CAZyme genes and functional interpretation very uncertain. In order to determine the strain capacity to break down a specific fiber, we linked CAZy genes to a given fiber or prebiotic. However, this representation has limitations because (i) the complexity and substrate diversity hardly fit the CAZyme databases and several families are displaying a wide phylogenetic diversity, such as amylase GH13 (as described by Stam et al., 2007) and (ii) certain families can be dedicated to/involved in different substrates (GH32 targeting inulin and levan as showed by Sheridan et al., 2015). Different authors reported different gene and substrate associations. As the gene content is then associated with functional capacities, it can have far-reaching consequences on the conclusions and on further applications or recommendations.

Which experimentations could help refine the pipeline? A major challenge toward quantifying the degree of redundancy of CAZymes will be to obtain more *in vitro* information on growth and CAZyme expression patterns. Up to now, there are still very few measurements of purified enzymes to pinpoint the specific substrate specificity of the CAZymes involved in the hydrolysis and fermentation of fiber or of the human gut mucus. We also modeled Firmicutes PULs (Supplementary Figure S5), but despite encouraging results, the scarcity of transcriptomics data available led us to focus on Bacteroidetes.

One way to circumvent these drawbacks or limits is to obtain massive large datasets performed in the same conditions, with strains for which the genomes are available. To improve FiberGrowth pipeline performance, a larger number of strains should be grown in the exact same conditions. This could be done using robots under anaerobic chamber, similar to the work by Zou et al. (2019), leading to > 6,000 isolated strains and 1,520 new genomes sequenced. Note that if more experimental data become available, it would also be possible to adopt a machine-learning approach to infer potential PUL rather than having a deterministic approach such as FiberGrowth.

Another way to increase our capacity to obtain large growth dataset arises from the recent advances in droplet microfluidic culture, which can increase the capacity and lower the cost of purified substrates, to screen for growth on fiber. Villa et al. (2020) recently demonstrated the potential of microfluidic droplet assays for comparing the growth rates and functions of individual bacterial strains isolated from gut microbial communities. This would be very useful in order to improve FiberGrowth pipeline on a large number of strains grown in the exact same conditions.

Interestingly, the authors also investigated how screening for GH with microfluidics could lead toward the differentiation of subjects, based on the “fiber profile” being metabolized by their microbiota. A limit of the characterization of a fiber-metabolizing potential per microbiota remains the complexity and modularity of the GH and PUL operons. Predicting the overall microbiome response to a specific fiber requires to account for the variability of GH protein structures. However, an accurate annotation of PUL seems difficult in metagenomics datasets. We already showed, using 40-kb *E. coli* fosmid libraries built from fecal samples or distal ileum mucosa, that glycosyl hydrolases were modular and subject to recent horizontal gene transfer, not just between phylogenetically close genomes but above the genus level (Tasse et al., 2010; Tauzin et al., 2020). A challenge remains to accurately annotate glycosyl hydrolase loci in metagenomic data, only feasible with long assembly with enough coverage.

Predicting individual response to prebiotic or fiber intake, we previously had demonstrated that the diversity of fiber-rich food items correlated with microbiome 16S rDNA diversity in young adults (Tap et al., 2015). Others have demonstrated that a 5-day regimen with a plant-based diet compared to animal product-based diet (David et al., 2013) increased microbial diversity. Mining existing large datasets such as iHMP2 (Proctor et al., 2019) can be of importance to find correlations between the breakdown of polymers from human origin, which have been linked to inflammatory bowel diseases. Those include not only human mucus and related compounds, such as heparin, but also human gangliosides. As a growing number of metagenomics datasets are being generated worldwide, correlations can be inferred between dietary intake and microbiome composition. However, most food questionnaires are not designed to provide indication on specific fiber. Interestingly, new AI-assisted tools for computing food images on smartphones may provide a more accurate picture of the ingested fiber content. There is still a lack of knowledge between a food element/items and the set of glycosyl hydrolases necessary for its breakdown and the subsequent production of beneficial metabolites through fermentation.

## Conclusion

The diversity of the CAZy gene families involved in the breakdown of glycans and the extreme modularity of the operons that encode them, sometimes at the strain level, offer a challenge to building PUL models that are generic for a given fiber. However, we demonstrated, for the first time, that a pipeline, combining automated genome screening and annotation of PULs, allowed us to build growth prediction models and measure their accuracies on Bacteroidetes available growth data. Some PUL models require optimization, while for levan, heparin, inulin, and arabinoxylan, FiberGrowth pipeline can compute growth estimations with TPR > 0.8 and FPR < 0.33 in 1 min on an unannotated genome.

This work also demonstrated that, despite advances in PUL bioinformatics screening and computing modeling, the lack of biochemical characterization of glycosyl hydrolases and PUL systems remains a main issue.

## Data Availability Statement

The original contributions presented in the study are included in the article/Supplementary Material, further inquiries can be directed to the corresponding author/s.

## Author Contributions

BC and CS designed and built the pipeline, performed all computing, and participated in the manuscript writing. FP supervised computing and assisted in the manuscript editing. ML was involved in the pipeline design and supervised the manuscript writing. All authors contributed to the article and approved the submitted version.

## Conflict of Interest

FP and CS work at Pendulum Therapeutics Inc. ML was member of the scientific advisory board for Pendulum Therapeutics. The remaining author declares that the research was conducted in the absence of any commercial or financial relationships that could be construed as a potential conflict of interest.

## Publisher’s Note

All claims expressed in this article are solely those of the authors and do not necessarily represent those of their affiliated organizations, or those of the publisher, the editors and the reviewers. Any product that may be evaluated in this article, or claim that may be made by its manufacturer, is not guaranteed or endorsed by the publisher.
