# Supplementary figures and images for: FiberGrowth Pipeline: A Framework Toward Predicting Fiber-Specific Growth From Human Gut Bacteroidetes Genomes
Source: Front Microbiol. 2021 Oct 6;12:632567. doi: 10.3389/fmicb.2021.632567 (PMC8527192; doi:10.3389/fmicb.2021.632567)

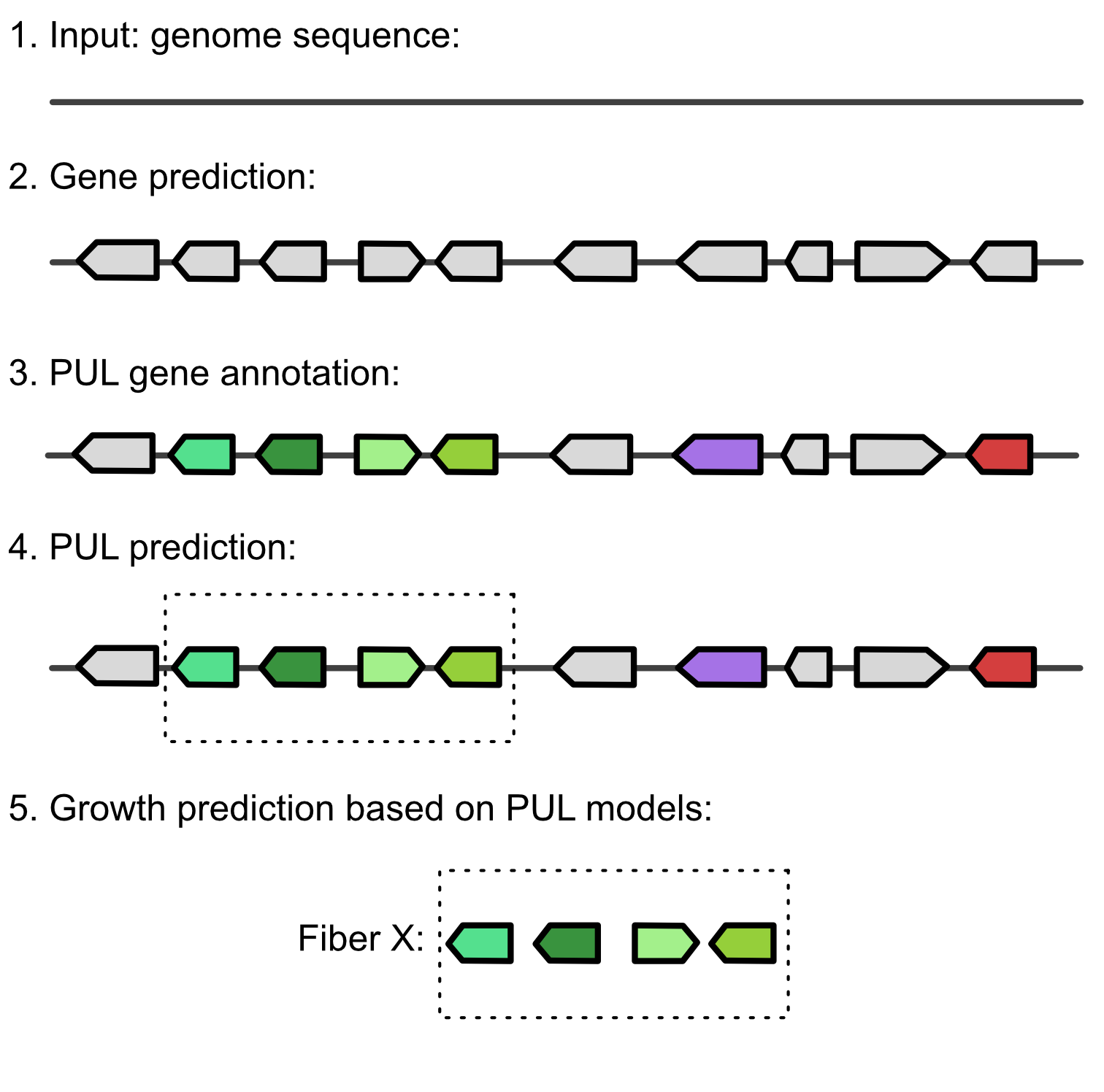

Supplement: Supplementary file 1 [file Data_Sheet_1.zip › Figure S0_Pipeline.png]

# Gene family

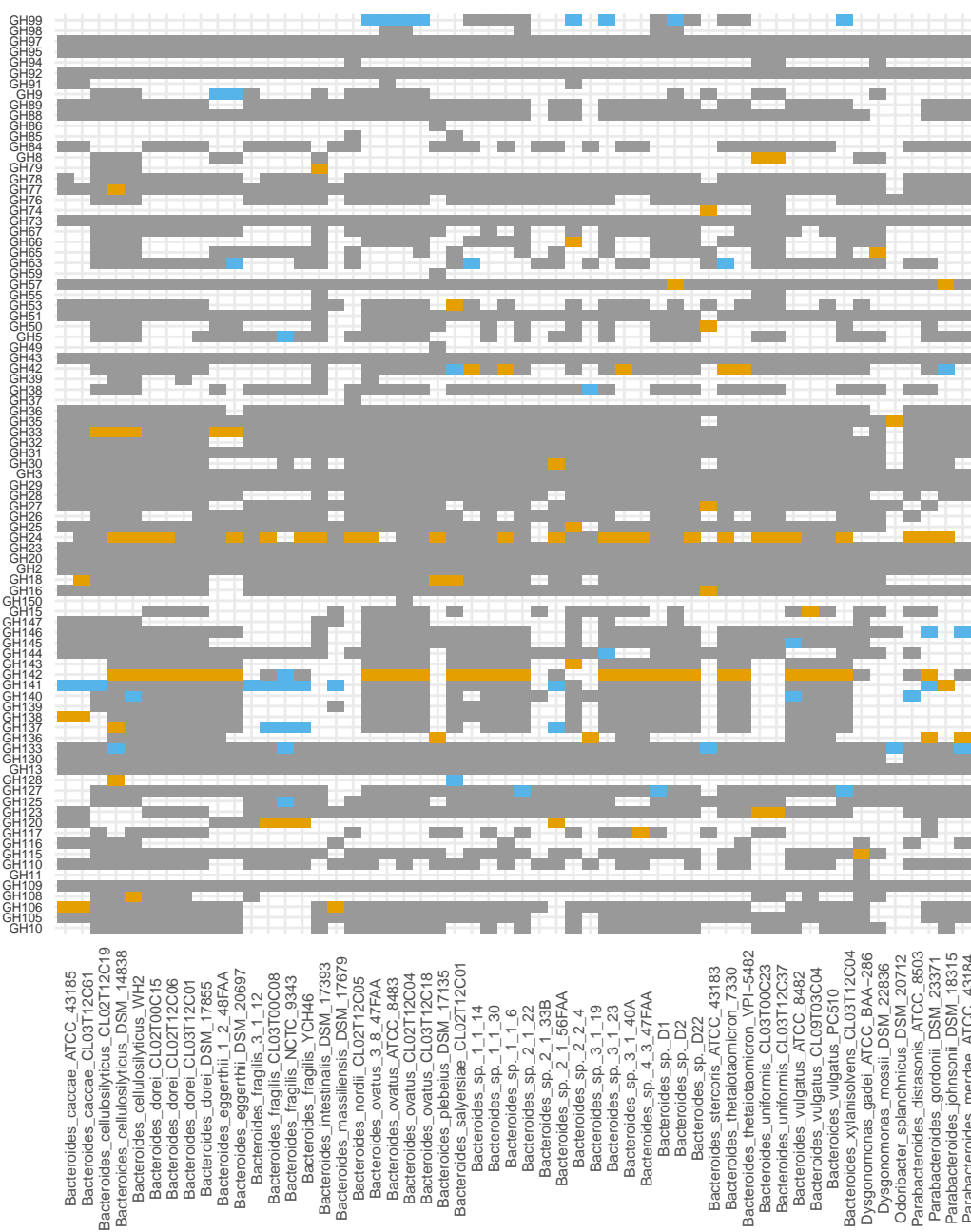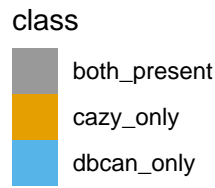

Supplement: Supplementary file 1 [file Data_Sheet_1.zip › Figure S1_detailed_CAZy_dbcan_annotation_comparison.pdf]

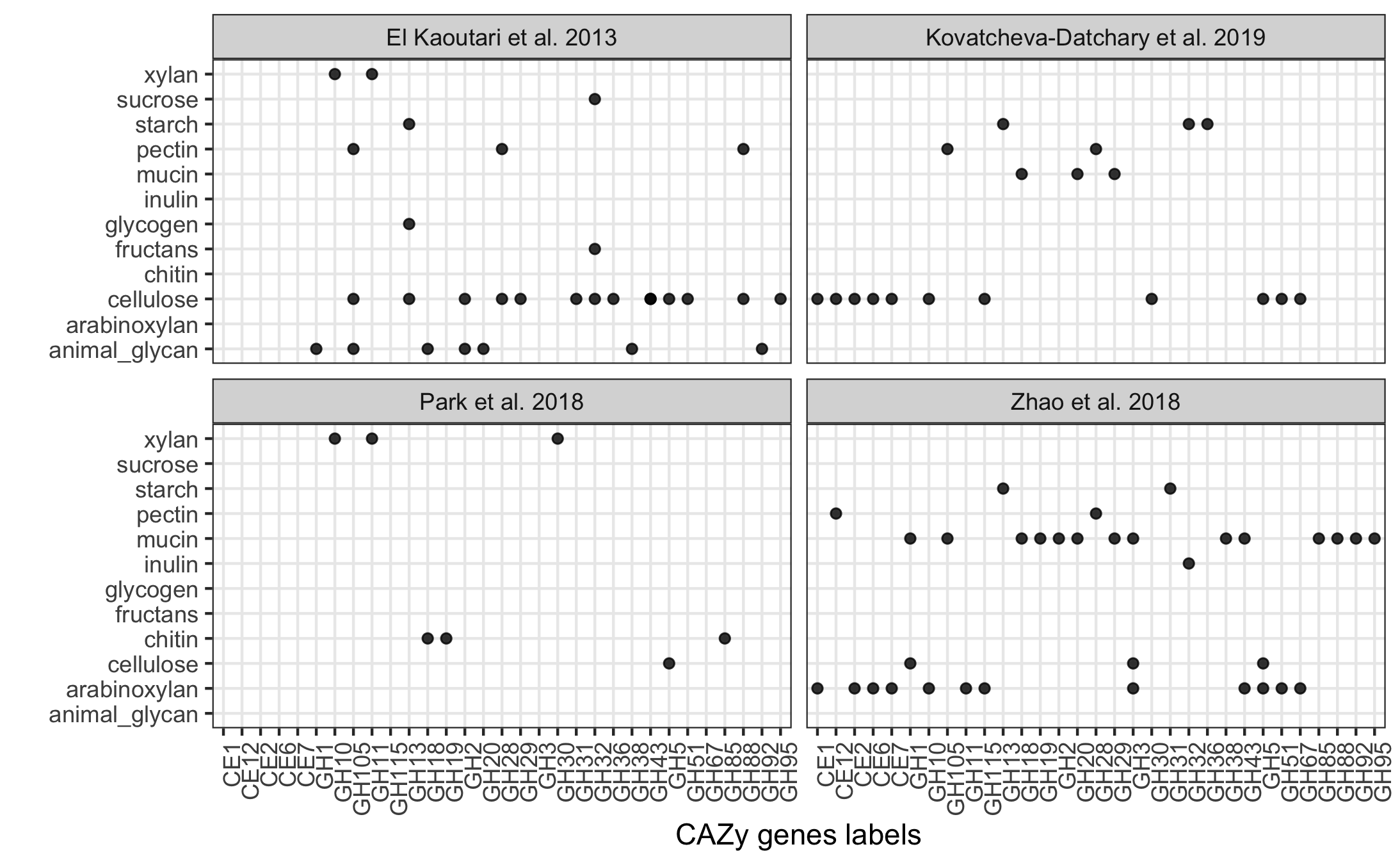

Supplement: Supplementary file 1 [file Data_Sheet_1.zip › Figure S2_GH Fiber litterature.png]

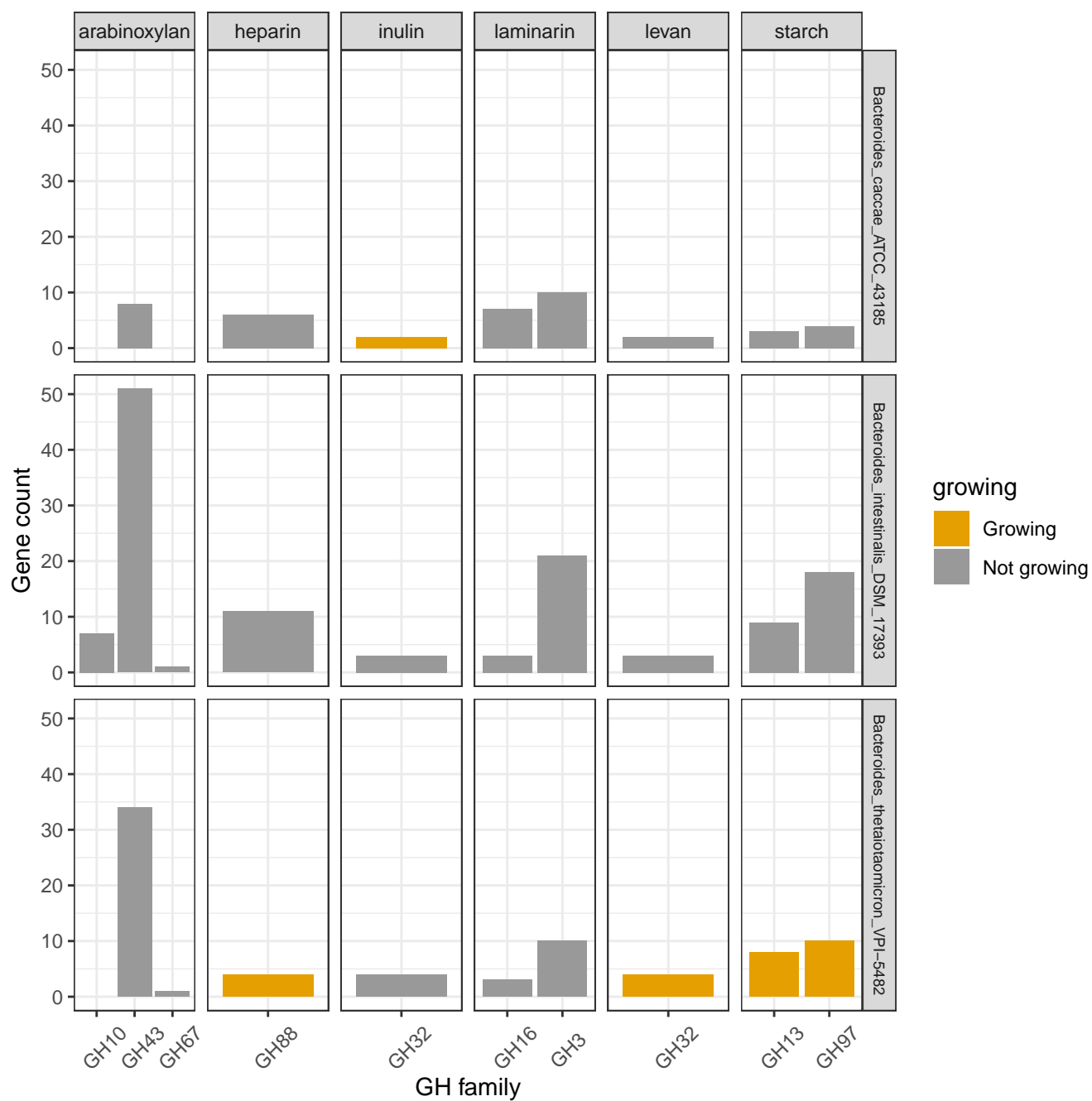

Supplement: Supplementary file 1 [file Data_Sheet_1.zip › Figure S3_GH_count_CAZy_growth.pdf]

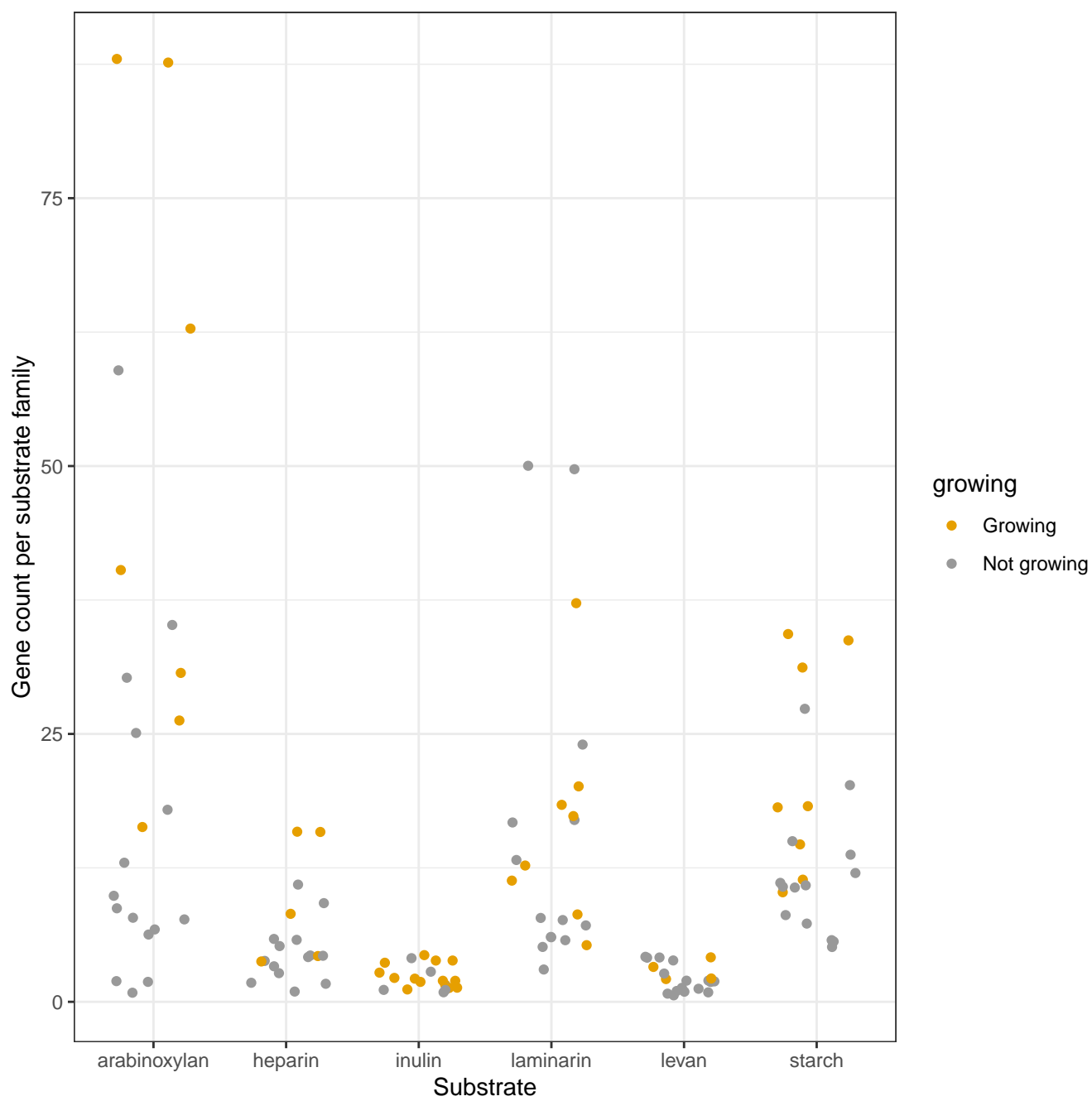

Supplement: Supplementary file 1 [file Data_Sheet_1.zip › Figure S4_GH_counts_and_growth.pdf]
